# Arginine-Mediated Liver Immune Regulation and Antioxidant Defense in Largemouth Bass (*Micropterus salmoides*): Multi-Omics Insights into Metabolic Remodeling During *Nocardia seriolae* Infection

**DOI:** 10.3390/antiox14060681

**Published:** 2025-06-03

**Authors:** Yu-Long Sun, Shuai-Liang Zhang, Feng-Feng Zhou, Yuan-Xin Qian, Yang He, Run-Zhe Zhang, Fen Dong, Qiang Chen, Han-Ying Xu, Ji-Teng Wang, Yu-Ting Deng, Tao Han

**Affiliations:** 1 Department of Aquaculture, Zhejiang Ocean University, Zhoushan 316022, China; 2022214@zjou.edu.cn (Y.-L.S.); goodhantao@gmail.com (T.H.); 2 Key Laboratory of Sichuan Province for Fishes Conservation and Utilization in the Upper Reaches of the Yangtze River, Neijiang Normal University, Neijiang 641000, China; 3 Key Laboratory of Fishery Drug Development of Ministry of Agriculture and Rural Affairs, Guangdong Provincial Key Laboratory of Aquatic Animal Immunology and Sustainable Aquaculture, Pearl River Fisheries Research Institute, Chinese Academy of Fishery Sciences, Guangzhou 510380, China

**Keywords:** *Micropterus salmoides*, arginine, glutathione, antioxidants, liver immune, transcriptome and metabolome analysis

## Abstract

The liver of fish is an essential metabolic organ that also serves an immune regulatory role. In this study, we constructed a model of largemouth bass (*Micropterus salmoides*) infected with *Nocardia seriolae* by injection to explore the immune and antioxidant functions of the liver. The results showed that *N. seriolae* infection caused severe pathological changes in the liver, including cell necrosis, granuloma formation, and leukocyte infiltration. The level of mRNA expression of immune-related genes in the liver was significantly increased 2 days post-infection. Moreover, the combined analysis of transcriptome and metabolome showed that *N. seriolae* infection markedly affected liver metabolism, including glutathione metabolism, arginine and proline metabolism, arachidonic acid metabolism, as well as starch and sucrose metabolism. Additionally, multiple key biomarkers were identified as involved in regulating responses to *N. seriolae* infection, including arginine, glutathione, *gpx*, *GST*, *PLA2G*, *GAA*, and *PYG*. To further elucidate the regulatory effects of arginine on the immune and antioxidant processes in the liver, primary hepatocytes were isolated and cultured. The results demonstrated that arginine supplementation significantly reduced the expression of LPS-induced apoptosis-related genes (*bax*, *cas3*, *cas8*, and *cas9*) by up to 50% while increasing the expression of antioxidant genes (*gpx*, *GST*) by up to 700% at 24 h. Through the analysis of metabolic changes and immune responses in the liver following *N. seriolae* infection, combined with in-vitro experiments, this study elucidated the anti-apoptotic and antioxidant effects of arginine, revealing the immune response mechanisms in fish liver and laying the groundwork for using nutritional strategies to improve fish health.

## 1. Introduction

The liver is the primary organ for metabolism. Nutrients absorbed by the body through the small intestine are transported to the liver [1]. In addition, the liver works as a physical barrier, filtering out harmful antigens and preventing them from invading the body [2]. The human liver is a vital immune tissue that contains a significant number of immune cell types [3]. In fishes, the liver not only regulates metabolism but also plays a critical part in immune regulation and antioxidant processes. Previous research has discovered that there are resident white blood cells (intrahepatic immune cells) in the liver of rainbow trout (*Oncorhynchus mykiss*), accounting for 15–29% of non-hepatocyte cells in the liver, forming an independently regulated immune cell population [4]. For example, the liver of rainbow trout is involved in immune regulation during viral hemorrhagic septicemia virus (VHSV) infection, with CD8α^+^ T-cells playing an important role [5]. Considering the important role of the liver in metabolism, we hypothesize that the strategy of balancing metabolism and immune function in the liver is crucial for the homeostasis of fish in the face of adverse external factors (e.g., environmental stress or pathogen infection).

The aquatic environment in which fish live can be subjected to toxins [6], heavy metal ions [7], and opportunistic pathogens [8,9], which pose a great threat to the survival of fish. Pathogen infection can trigger apoptosis in host cells and induce an inflammatory response against the invasion of the pathogen [10,11]. *Nocardia*, a Gram-positive bacterium, can infect a variety of marine and freshwater fishes [12]. After invading the host, the pathogen mainly infects organs such as the liver, head, kidney, and spleen, forming granulomatous nodules in the tissues, which is also a typical clinical sign of *Nocardia* infection [13]. Nodules in the fish are a particular structure formed by the interplay of *Nocardia* and host cell-mediated immune response [14,15]. In vitro, *N. seriolae* can induce apoptosis of macrophages of largemouth bass (*Micropterus salmoides*); however, with prolonged infection time, the pathogen can also inhibit the apoptosis of macrophages to increase intracellular survival [16]. *M. salmoides* is one of China’s important economically farmed species, with aquaculture production increasing year by year, reaching 880,000 tons in 2023 [17]. However, outbreaks of *N. seriolae* infection have led to severe economic losses, with reported mortality rates ranging from 20% to 60% at affected farms [18,19]; yet, the pathogenic mechanism of *N. seriolae* infection in *M. salmoides* remains unclear, necessitating further investigation.

Transcriptome analysis, a high-throughput transcriptome sequencing approach, provides complete genome sequences and gene expression information, enabling a comprehensive study of the cell’s transcriptional profile [20]. Using transcriptomics technology, researchers analyzed the immune response of largemouth bass spleen [21] and peripheral blood of hybrid snakehead (*Channa maculata* ♀ × *Channa argus* ♂) [22] after *N. seriolae* infection. Previous studies have reported the important role of N-acetylglucosamine in anti-*Streptococcus iniae* infection by analyzing the metabolic profile of Nile tilapia (*Oreochromis niloticus*) liver [23]. In recent years, an increasing number of studies have employed the joint analysis of the transcriptome and metabolome to explore the response strategies of aquaculture animals to heavy metal stress, nutrition change, or pathogen infection [9,24,25].

Nutritional strategies have garnered increasing interest in recent years for their potential to enhance fish immunity and improve resistance to pathogen infections and oxidative stress [26,27,28,29,30]. Arginine, a precursor for the synthesis of nitric oxide (NO), polyamines, creatine, and guanidinoacetate, has been demonstrated to enhance immune function and mitigate cellular oxidative stress in crustaceans and juvenile blunt snout bream (*Megalobrama amblycephala*) [31,32]. Its capacity to alleviate oxidative stress-induced cellular damage, thereby enhancing antioxidant capacity [28,32], underscores its importance as an immunonutrient [33], leading to considerable attention regarding its role in fish’s immunity. Furthermore, arginine and its metabolites play critical roles in physiological processes, including promoting cell proliferation, enhancing antioxidant defenses, and regulating energy metabolism [34,35]. With respect to inflammation, arginine can modulate the Toll-like receptor-NF-κB signaling pathway, thereby influencing the expression and activity of various inflammatory factors and exerting immunomodulatory effects [36]. Specifically, it inhibits the expression of intestinal inflammatory factors induced by lipopolysaccharides (LPS), thus exhibiting anti-inflammatory properties [37]. For example, dietary arginine supplementation significantly reduced inflammation and apoptosis triggered by LPS stimulation in Jian carp (*Cyprinus carpio* var. Jian) [37]. Moreover, in the context of repairing oxidative stress-induced damage, arginine enhances the activity of antioxidant enzymes, such as glutathione-S-transferase (GST), glutathione peroxidase (gpx), and superoxide dismutase (SOD), mitigating the impact of oxidative stress on the physical barriers of fish [28,32,38] and increasing antioxidant enzyme levels in the intestines and muscles of grass carp (*Ctenopharyngodon idella*) to exert antioxidant effects [39].

This study investigates the immunomodulatory and antioxidant mechanisms within largemouth bass liver following *N. seriolae* infection. While single-omics approaches, such as transcriptomics, effectively reveal gene expression changes, they fail to fully unravel critical regulatory pathways. Metabolomics, relying on liquid chromatography-mass spectrometry (LC-MS), enables the characterization and quantification of metabolites in biological samples. Although metabolomic profiles reflect phenotypic states, they cannot elucidate the genetic mechanisms underlying these phenotypes [24,25]. Therefore, the joint analysis of transcriptome and metabolome was used to analyze the metabolic mechanisms in the liver after infection with *N. seriolae*. By employing pathological analysis, transcriptional profiling of immune and antioxidant-related genes, multi-omics integration, and an in-vitro LPS-induced hepatocyte inflammation model, this research explores arginine-mediated regulatory mechanisms during *N. seriolae* infection. Abbreviations and full names are provided in the Section of Backmatter.

## 2. Materials and Methods

### 2.1. Fish Farming

Largemouth bass (~10 g) were obtained from Chia Tai Aquatic Products Co., Ltd. (Huzhou, China). Fish were fed twice daily and maintained for two weeks to adjust to the experimental conditions. The experiments strictly adhered to the guidelines of the Experimental Animal Welfare Ethics Committee of Zhejiang Ocean University [40].

### 2.2. Infection Experiment and Sample Collection

The strain of *N. seriolae* (NK20211208) was generously provided by Yuting Deng at the Pearl River Fisheries Research Institute, Chinese Academy of Fishery Sciences, Guangzhou, China. According to a previous experiment [8], fish were intraperitoneally injected with 1 × 10^4^ CFU/mL of *N. seriolae* at a volume of 100 µL. The control group of fish was injected with 100 μL of saline. Liver tissues of the *M. salmoides* were dissected for histological examination and transcription-level analysis of genes at 1, 2, 4, 7, and 14 days post-infection (dpi).

### 2.3. Histological Analysis

Liver tissues were fixed in 4% paraformaldehyde for 24 h, followed by a 5-min rinse under running water to remove residual paraformaldehyde from the tissue. After gradient dehydration with ethanol, the tissue was embedded in paraffin. Following paraffin sectioning, hematoxylin and eosin (H&E) staining was performed. The images were captured with a microscope after the slides were sealed with neutral balsam.

### 2.4. RNA Extraction, Reverse Transcription, and Quantitative PCR (qPCR)

Total RNA extraction from liver samples was performed using the TRIzol reagent (Thermo Fisher Scientific, Inc., Waltham, MA, USA) method [8]. Briefly, the tissue was homogenized in 1 mL of TRIzol for one minute, followed by centrifugation to collect the supernatant. The supernatant was mixed with 200 µL of chloroform and incubated on ice for 5 min before centrifugation. The supernatant was then transferred to a new 1.5 mL Eppendorf (EP) tube. After adding an equal volume of isopropanol and mixing, the solution was incubated on ice for 10 min. Following centrifugation, the supernatant was discarded, and the pellet was then washed with 75% ethanol and finally dissolved in 0.1% (*v*/*v*) diethyl pyrocarbonate (DEPC)-treated water. Reverse transcription to synthesize cDNA was conducted according to the HiFiScript cDNA Synthesis Kit (CoWin Biosciences, Jiangsu Cowin Biotech Co., Ltd., Taizhou, China) instructions. qPCR experiments were performed using the SYBR qPCR Master Mix kit (Vazyme Biotech Co., Ltd., Nanjing, China). The reaction conditions were as follows: 95 °C for 3 min, followed by 40 cycles of 95 °C for 10 s, 58 °C for 10 s, and 72 °C for 20 s. The primer sequences in the experiments are listed in Supplementary Table S1. *β-actin* was used as the reference gene, and gene expression was calculated using the −ΔΔCt method [41].

### 2.5. Transcriptome Analysis

Following the protocol of a previous study [24], transcriptomic analysis was conducted. In brief, liver samples from both the control group and those infected for 7 days (three samples per group) were sent to Biomarker Technologies Co., Ltd. (Beijing, China) for transcriptome analysis. RNA was extracted from the samples using TRIzol reagent (Thermo Fisher Scientific, Inc.), followed by the synthesis of the first and second strands of cDNA. Sequencing was performed using an Illumina NovaSeq 6000 (Illumina, Inc., San Diego, CA, USA). After sequencing, raw data processing and bioinformatics analysis were conducted using the BMKCloud platform (www.biocloud.net). The raw sequencing data underwent quality filtering to remove low-quality reads. Specifically, clean data were obtained by eliminating reads containing adapters, poly-N sequences, or low-quality bases from the raw data. Additionally, Q20, Q30, GC content, and sequence duplication levels of the clean data were calculated. The Q30 base percentage for all samples exceeded 92.89%, ensuring data reliability. The clean data were aligned to the largemouth bass reference genome (GCF_014851395.1_ASM1485139v1) using HISAT2 v2.0.4 (https://www.hpc.cineca.it/systems/software/hisat2/, accessed on 10 May 2024). Transcript assembly and expression quantification were performed using StringTie v2.2.1 (https://ccb.jhu.edu/software/stringtie/, accessed on 10 May 2024), which employs a maximum flow algorithm for transcript identification. Gene expression levels were normalized using FPKM to ensure accurate quantification [42]. Differential gene expression analysis was conducted using DESeq2 v1.30.1 [43]. Genes with an adjusted *p*-value < 0.01 and a fold change ≥2 were designated as differentially expressed. The identified genes were annotated and identified via Gene Ontology (GO, http://www.geneontology.org/) and Kyoto Encyclopedia of Genes and Genomes (KEGG, http://www.genome.jp/kegg/, accessed on 10 May 2024) assessments using OmicShare tools (https://www.omicshare.com/, accessed on 10 May 2024).

### 2.6. Untargeted Metabolome Analysis

The liver samples from the control and the experimental group infected for 7 days (6 samples per group) were sent to Biomarker Technologies Co., Ltd. (Beijing, China) for metabolomic analysis. After extracting the metabolites from the tissues, they were analyzed with liquid chromatography-mass spectrometry (Waters Acquity I-Class PLUS UPLC coupled to a Waters XEVO G2-XS quadrupole time-of-flight (QTOF) mass spectrometer; Waters Co., Milford, MA, USA). The raw data were acquired using MassLynx V4.2 (Waters Co.) and processed through Progenesis QI software v2.3 (https://www.nonlinear.com/), which performed peak extraction, peak alignment, and other data processing steps. Metabolite identification was conducted using Progenesis QI software, the METLIN online database, public databases, and the self-built library of Biomarker Technologies [44,45,46]. Theoretical fragment identification was performed with a parent ion mass deviation within 100 ppm and a fragment ion mass deviation within 50 ppm [46]. Subsequently, compound identification and differential analysis were carried out using the KEGG, HMDB (https://hmdb.ca/), and Lipidmaps (https://lipidmaps.org/) databases. Fold changes were calculated and compared, and *T*-tests were applied to determine the statistical significance of differential compounds (*p*-value < 0.05). To further refine the analysis, OPLS-DA (orthogonal projections to latent structures-discriminant analysis) was conducted using the ropls v1.6.2 package in R 3.6.1 [47], and variable importance in projection (VIP) values were computed. Metabolites showing significant differences between groups were screened based on VIP > 1 and *p* < 0.05. The KEGG pathway of differential metabolites was analyzed using OmicShare tools platform (https://www.omicshare.com/, accessed on 10 May 2024).

### 2.7. Joint Analysis of Transcriptome and Metabolome

The OmicShare online analysis tool was used for the joint analysis of metabolome and transcriptome. The co-enriched pathways in the transcriptome and metabolome KEGG pathways were plotted based on the number of differentially expressed genes (DEGs) and the *p*-value of differentially expressed metabolites (DMs) in the liver. Additionally, a Sankey diagram of DEGs-DMs-pathways was created based on the correlation of DEGs and DMs. A gene-metabolite correlation heatmap was constructed based on the abundance of DEGs and DMs. A most significantly enriched DEGs and DMs interaction network diagram was drawn based on the correlation heatmap. The KEGG mapping results of DEGs and DMs were visualized using the pathview online tool v1.1.6.

### 2.8. Isolation and Culture of Hepatocytes

Liver tissue was excised, fragmented, and washed repeatedly. It was then incubated in an antibiotic medium (Dulbecco’s Modified Eagle Medium (DMEM), 2% penicillin-streptomycin (PS), 12.5 μg/mL amphotericin B, 0.5 mg/mL gentamicin sulfate) for 2 h in a cell culture incubator. Subsequently, the sterile tissue fragments were inoculated into T25 cell culture flasks and cultured under adherent conditions until cell migration was observed (28 °C, 5% CO_2_). The adherent cells were then trypsinized and seeded into 6-well plates for overnight culture. To eliminate the confounding effects of arginine, the fetal bovine serum (FBS) concentration in the modified DMEM was set to 5%, consistent with previous studies [40]. Hepatocyte viability exceeded 90% based on morphological evaluation, ensuring suitability for arginine supplementation experiments. Hepatocytes were then incubated with varying concentrations of arginine (0 mM for the arginine-deficient group and 0.4 mM for the arginine-supplemented group) to investigate the in-vitro effects of arginine on hepatocyte immune function. In this study, 0.4 mM represented the standard arginine concentration in DMEM. Arginine (0.4 mM) and LPS (10 μg/mL, Solarbio, Beijing, China) were added to the culture plates in four treatment groups: Negative control: Arg^−^ LPS^−^ (base medium); Arginine treatment: Arg^+^ LPS^−^; LPS challenge: Arg^−^ LPS^+^; Combined treatment: Arg⁺ LPS⁺. Cells were harvested at 3, 6, 12, and 24 h post-incubation (hpi) for analysis of the expression of key immune and antioxidant-related genes. Primer sequences used for qRT-PCR analysis, including *β-actin* as the reference gene, are listed in Supplementary Table S1.

## 3. Results

### 3.1. Pathological Changes and Gene Expression of Liver

**Figure 1:**
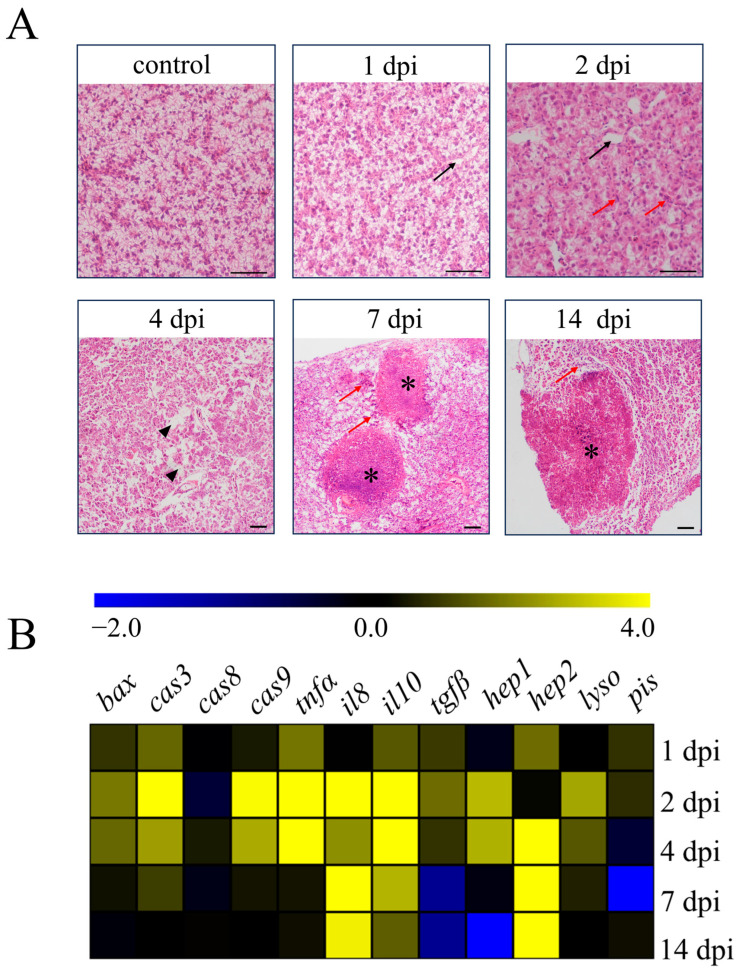
Pathological changes and transcription levels of immune-related genes in the liver of largemouth bass. (**A**) H&E staining of liver sections at different time points after *N. seriolae* infection (1 dpi, 1 day post-infection, 2 dpi, 2 days post-infection, 4 dpi, 4 days post-infection, 7 dpi, 7 days post-infection, 14 dpi, 14 days post-infection, black arrow indicates tissue edema, the red arrow indicates leukocytes infiltrating, black triangle indicates cell necrosis and abscission, and asterisk indicates granuloma, scale bar, 50 μm). (**B**) Effects of *N. seriolae* infection on the mRNA expression of genes associated with apoptosis, inflammation, and antibacterial function. *bax*, *bcl-2-associated x protein*; *cas3/8/9*, *caspase-3/8/9*; *tnfa*, *tumor necrosis factor alpha*; *il8/il10*, *interleukin-8/10*; *tgfβ*, *transforming growth factor beta*; *hepl/hep2*, *hepcidin-1/2*; *lyso*, *lysozyme*; *pis, piscidin*.

*N. seriolae* infection causes high mortality in farmed fish, with typical symptoms of infected fish including the presence of numerous white nodules in visceral organs such as the liver, spleen, and head kidney [13]. In our study, we found a large number of marked white nodules in the abdominal cavity, liver, and spleen of the infected fish (Supplementary Figure S1A). Then, we examined the pathological changes in the liver tissues of largemouth bass (*M. salmoides*) following infection with *N. seriolae* at different time points (Figure 1A). By H&E staining, the liver showed edema and vacuolation (black arrows) at 1 dpi compared with the control group. At 2 dpi, the inflammation of liver tissues further intensified, with numerous leukocytes infiltrating (red arrows) and aggravated tissue edema (black arrows). Extensive cell necrosis and abscission (black triangles) in the liver could be observed at 4 dpi, and obvious granulomatous (asterisks) could be observed with numerous leukocytes distributed around the granulomatous (red arrows) at 7 and 14 dpi (Figure 1A). Concurrently, we examined the transcription levels of apoptotic genes, inflammation-related genes, and antibacterial genes in the liver following *N. seriolae* infection (Figure 1B). The apoptotic genes *bcl-2-associated x protein* (*bax*), *caspase-3* (*cas3*), and *caspase-9* (*cas9*) were primarily expressed at peak levels at 2 dpi, except for the *caspase-8* (*cas8*) gene. The expression of the pro-inflammatory gene *tumor necrosis factor-alpha* (*tnfα*) increased from 1 dpi until 4 dpi, while *interleukin-8* (*il8*) showed increased expression from 2 dpi and continued until 14 dpi.

Expression of the anti-inflammatory gene *interleukin-10* (*il10*) was elevated markedly from 2 dpi until 7 dpi, and *transforming growth factor beta* (*tgfβ*) showed increased expression only at 2 dpi. The antibacterial gene *hepcidin-1* (*hep1*) showed the highest expression at 2 dpi, while the gene *hepcidin-2* (*hep2*) showed increased expression from 4 dpi and continued until 14 dpi. The transcription level of the *lysozyme* (*lyso*) gene increased at 2 dpi and 4 dpi. However, the *piscidin* (*pis*) gene did not show a significant change (Figure 1B).

### 3.2. DEG Analyses in the Liver Following N. seriolae Infection

**Figure 2:**
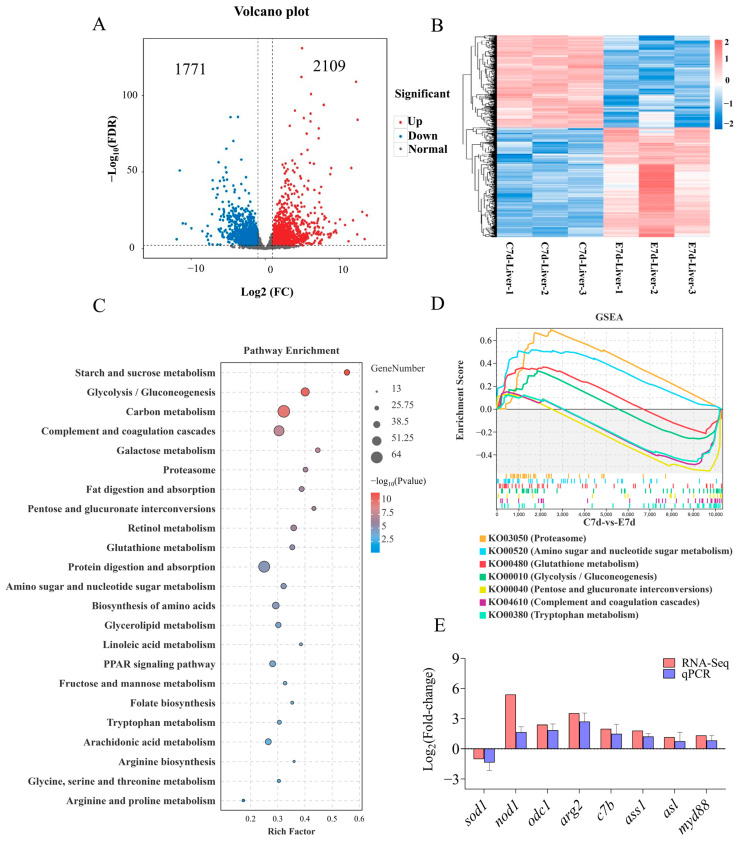
Transcriptome analysis of the DEGs in the liver of largemouth bass following *N. seriolae* infection (n = 3). (**A**) volcano plot. (**B**) heat map. The C7d-Liver represents the control group, and E7d-Liver represents the experimental group infected with *N. seriolae*. C7d-Liver 1/2/3 or E7d-Liver 1/2/3 represents three biological replicates. (**C**) KEGG pathway enrichment analysis bar diagram obtained from the transcriptome. The blue and red dots indicate notably downregulated and upregulated genes, respectively; the grey dot represents no significant change. (**D**) Gene Set Enrichment Analysis (GSEA) for the KO terms related to Figure 2C. (**E**) The accuracy of transcriptome data was verified using qPCR. *sod1*, *superoxide dismutase 1*; *nod1*, *nucleotide-binding oligomerization domain-containing protein 1*; *odc1*, *ornithine decarboxylase 1*; *arg2*, *arginase 2*; *c7b*, *complement component 7b*; *ass1*, *argininosuccinate synthase 1*; *asl*, *argininosuccinate lyase*; *myd88*, *myeloid differentiation primary response 88*. The results are presented as the means ± SD.

Transcriptome results showed that *N. seriolae* elicited significant changes in the transcription levels of 3880 genes in the liver, with 1771 genes significantly down-regulated and 2109 genes significantly upregulated (Figure 2A,B). KEGG pathway analysis of DEGs showed that the enriched pathways mainly included starch and sucrose metabolism, glycolysis/gluconeogenesis, as well as complement and coagulation cascades (Figure 2C). In addition, Gene Set Enrichment Analysis (GSEA) results showed significant differences in the processes of proteasome, amino sugar and nucleotide sugar metabolism, glutathione metabolism, glycolysis/gluconeogenesis, pentose and glucuronate interconversions, complement and coagulation cascades, and tryptophan metabolism (Figure 2D, Supplementary Figure S2). To validate the accuracy of the transcriptome results, the mRNA expression levels of 8 genes were examined using the qPCR method. The qPCR results showed that the expression patterns of genes were similar to those of the transcriptome, indicating the reliability of the transcriptome data (Figure 2E).

### 3.3. Change of Metabolome Profiles in the Liver Following N. seriolae Infection

**Figure 3:**
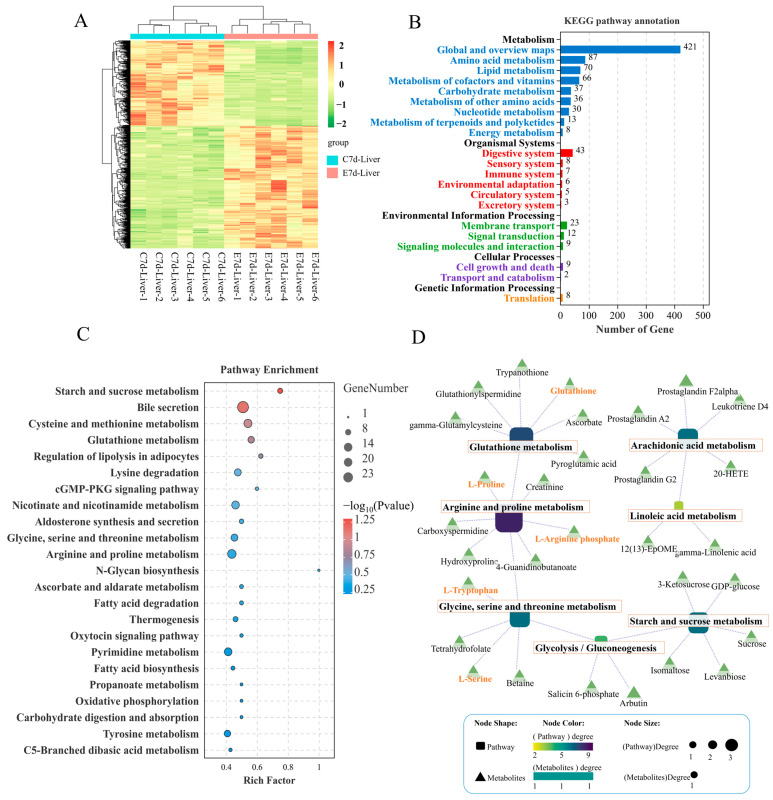
Metabolome analysis of the differential expressed metabolites (DMs) in the liver of largemouth bass from the control and *N. seriolae* infection groups (n = 6). (**A**) Heat map, (**B**) KEGG pathway annotation bar diagram, and (**C**) KEGG pathway enrichment analysis bar diagram of the DMs in the liver of largemouth bass infected with *N. seriolae* at 7 dpi. (**D**) The regulatory network between key metabolic pathways and DMs is visualized, where squares represent pathways and triangles represent metabolites. The closer the pathway color is to purple, the greater its importance within the metabolic regulatory network.

According to the VIP (variable importance in the projection) scores of the OPLS-DA (orthogonal projections to latent structures-discriminant analysis), results showed that infection of *N. seriolae* caused metabolic disturbances in the liver, with 945 metabolites significantly upregulated and 666 significantly downregulated (Figure 3A). Functional annotation of these DMs (differentially expressed metabolites) revealed that 87 metabolites in amino acid metabolism pathways were enriched, 70 metabolites in lipid metabolism pathways were enriched, and 37 metabolites were enriched in carbohydrate metabolism pathways (Figure 3B). Further KEGG pathway enrichment analysis of the DMs showed that the enriched pathways mainly included starch and sucrose metabolism, cysteine and methionine metabolism, glutathione metabolism, and arginine and proline metabolism (Figure 3C). The construction of a regulatory network integrating DMs and metabolic pathways revealed a close interconnection within an overall network of amino acid, carbohydrate, and lipid energy metabolism. This network encompasses pathways such as glutathione metabolism, arginine and proline metabolism, glycine, serine, and threonine metabolism, glycolysis/gluconeogenesis, starch and sucrose metabolism, arachidonic acid metabolism, and linoleic acid metabolism. Key DMs identified include glutathione, L-proline, L-arginine phosphate, L-tryptophan, and L-serine (Figure 3D). These core DMs are anticipated to play a crucial role in the liver’s defense against *N. seriolae* infection.

### 3.4. Joint Analysis of Transcriptome and Metabolome

**Figure 4:**
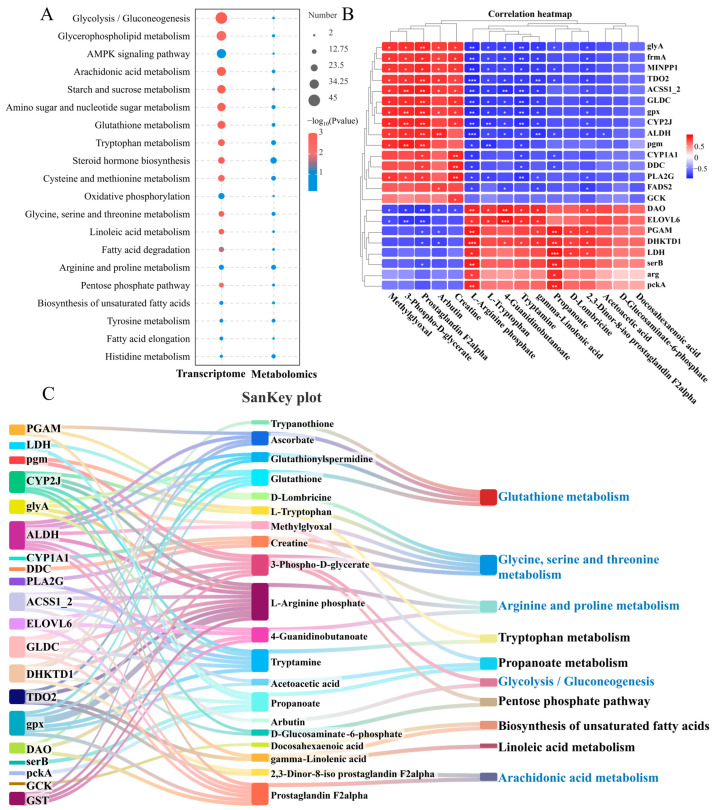
Joint analysis of DEGs and DMs in the liver of largemouth bass from the control and *N. seriolae* infection groups. (**A**) Co-annotated KEGG pathway of the transcriptome and metabolome. The left column represents the transcriptome, and the right column represents the metabolome. (**B**) Correlation heatmap of DEGs and DMs. The red or blue squares illustrate the positive or negative correlation between DEGs and DMs, respectively. Asterisks in the boxes indicate a significant correlation between DEGs and DMs, and a number of asterisks indicate significance levels: * for 0.01 < *p* < 0.05, ** for 0.001 < *p* < 0.01, and *** for *p* ≤ 0.001. (**C**) SanKey plot presenting the correlation of DEGs, DMs, and pathways in the liver of largemouth bass. The left boxes represent the key genes involved in the network, the middleboxes represent metabolites, and the right boxes represent the metabolic pathways.

By joint analysis of the transcriptome and metabolome, the common enrichment pathways for DEGs and DMs are glycolysis/gluconeogenesis, glycerophospholipid metabolism, arachidonic acid metabolism, starch and sucrose metabolism and glutathione metabolism. These pathways mainly include amino acid, lipid, and carbohydrate metabolism (Figure 4A). To better understand the relationship between these DEGs and DMs after *N. seriolae* infection, the study examined their correlations and visualized them using correlation heatmaps and a Sankey diagram. The correlation heatmap clustered genes and metabolites with similar coefficients, shedding light on their interactions (Figure 4B). Meanwhile, a DEGs-DMs-pathways correlation Sankey diagram was constructed (Figure 4C). Where there are interconnections between DEGs, DMs, and metabolic pathways post-infection, the Sankey diagram connects them, showing potential biological information flow (Figure 4C). The enriched pathways in the correlation analysis included amino acid metabolism (glutathione metabolism, glycine, serine, and threonine metabolism, arginine and proline metabolism, and tryptophan metabolism), lipid metabolism (biosynthesis of unsaturated fatty acids, linoleic acid metabolism, and arachidonic acid metabolism), and carbohydrate metabolism (glycolysis/gluconeogenesis).

### 3.5. Amino Acid-Related Metabolic Changes in the Liver

**Figure 5:**
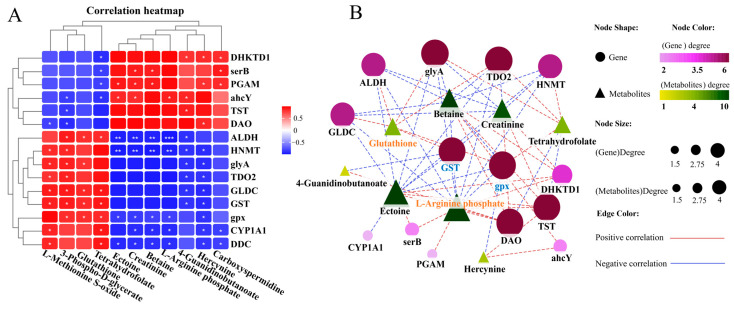

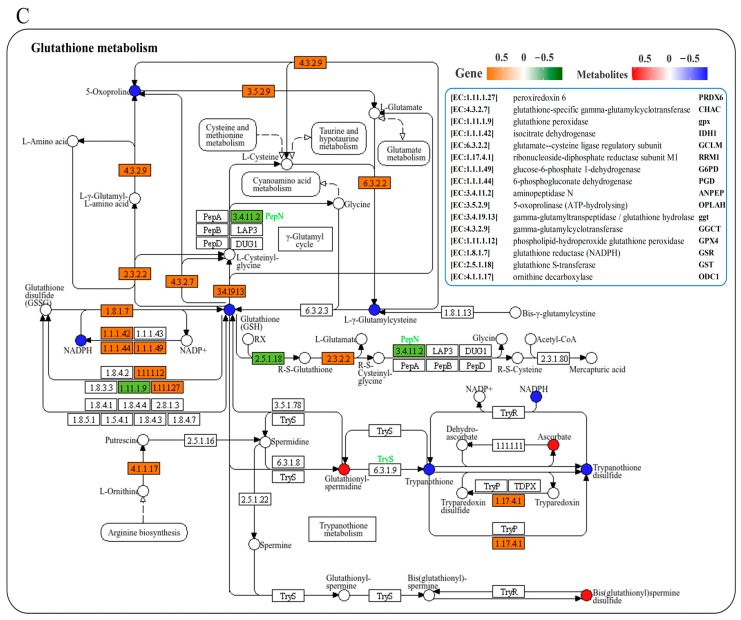

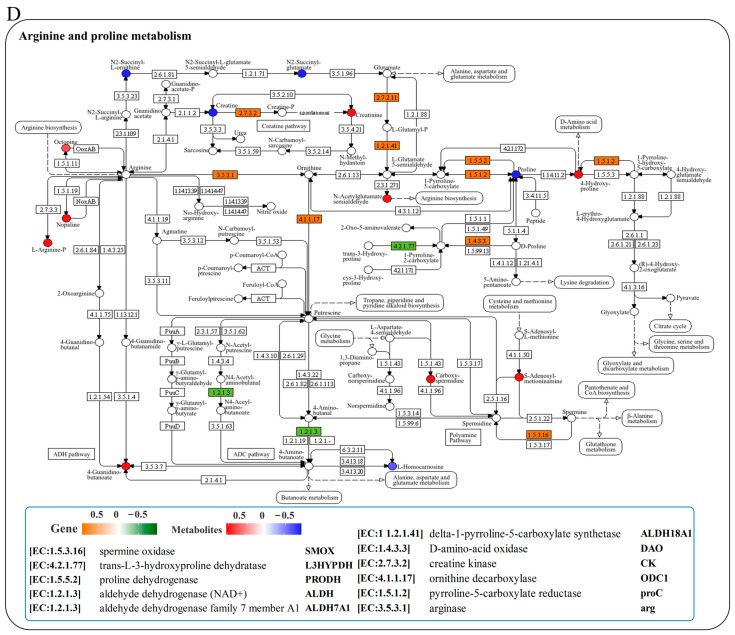
Joint analysis of DEGs and DMs in amino-acid metabolic pathways. (**A**) Correlation heatmap of DEGs and DMs. The red or blue squares illustrate the positive or negative correlation between DEGs and DMs, respectively. Asterisks in the boxes indicate a significant correlation between DEGs and DMs, and a number of asterisks indicate significance levels: * for 0.01 < *p* < 0.05, ** for 0.001 < *p* < 0.01, and *** for *p* ≤ 0.001. (**B**) Interaction network analysis of the most significantly enriched DEGs and DMs in amino acid metabolic pathways in the liver of largemouth bass. The orange dotted lines show a positive correlation between DEGs and DMs, while the blue dotted lines reveal a negative correlation between DEGs and DMs. (**C**) The DEGs and DMs in the glutathione metabolism mapped to the KEGG pathway diagram. (**D**) The DEGs and DMs in the arginine and proline metabolism pathway mapped to the KEGG pathway diagram. The orange or green box indicated significant upregulation or downregulation of the gene, respectively. The red or blue circle represented significant upregulation or downregulation of the metabolite, respectively.

We conducted a joint analysis to describe the correlation between gene expression and metabolites related to amino-acid metabolism in the liver after *N. seriolae* infection. The correlation heatmap results showed the presence of a correlation network consisting of 15 key genes and 11 DMs in the liver, which changed similarly or oppositely concurrently (Figure 5A). The results of the interaction network diagram showed the presence of an amino-acid metabolism regulatory network consisting of 14 core gene nodes, including *gpx*, *GST*, *CYP1A1* (*cytochrome p450 family 1 subfamily a1*), *DHKTD1* (*2-oxoadipate dehydrogenase e1 component*), *ALDH* (*aldehyde dehydrogenase* (NAD+)), *HNMT* (*histamine n-methyltransferase*), *ahcY* (*adenosylhomocysteinase*), *glyA* (*glycine hydroxymethyltransferase*), *serB* (*phosphoserine phosphatase*), *GLDC* (*glycine dehydrogenase*), *TDO2* (*tryptophan 2,3-dioxygenase*), *TST* (*thiosulfate/3-mercaptopyruvate sulfurtransferase*), *DAO* (*d-amino-acid oxidase*), and *PGAM* (*2,3-bisphosphoglycerate-dependent phosphoglycerate mutase*), as well as 8 core metabolite nodes (glutathione (GSH), ectoine, tetrahydrofolate, betaine, 4-guanidinobutanoate, creatinine, hercynine, L-arginine phosphate) (Figure 5B). Among them, glutathione, L-arginine phosphate, ectoine, tetrahydrofolate, creatinine and betaine showed the strongest correlation with other genes and metabolites (Figure 5B). Further, KEGG mapping analysis indicated that *N. seriolae* infection affected glutathione metabolism (Figure 5C), arginine and proline metabolism (Figure 5D) and glycine, serine, and threonine metabolism (Supplementary Figure S3). Specifically, the constructed regulatory network for glutathione metabolism revealed a downregulated pattern in key genes *gpx* and *GST*, both crucial for glutathione synthesis and redox homeostasis. Notably, the significant suppression of the critical differential metabolite GSH correlated with the decreased expression of *gpx* and *GST* (Figure 5C), indicating a weakened antioxidant defense mechanism in the liver following pathogen infection.

### 3.6. Lipid-Related Metabolic Changes in the Liver

**Figure 6:**
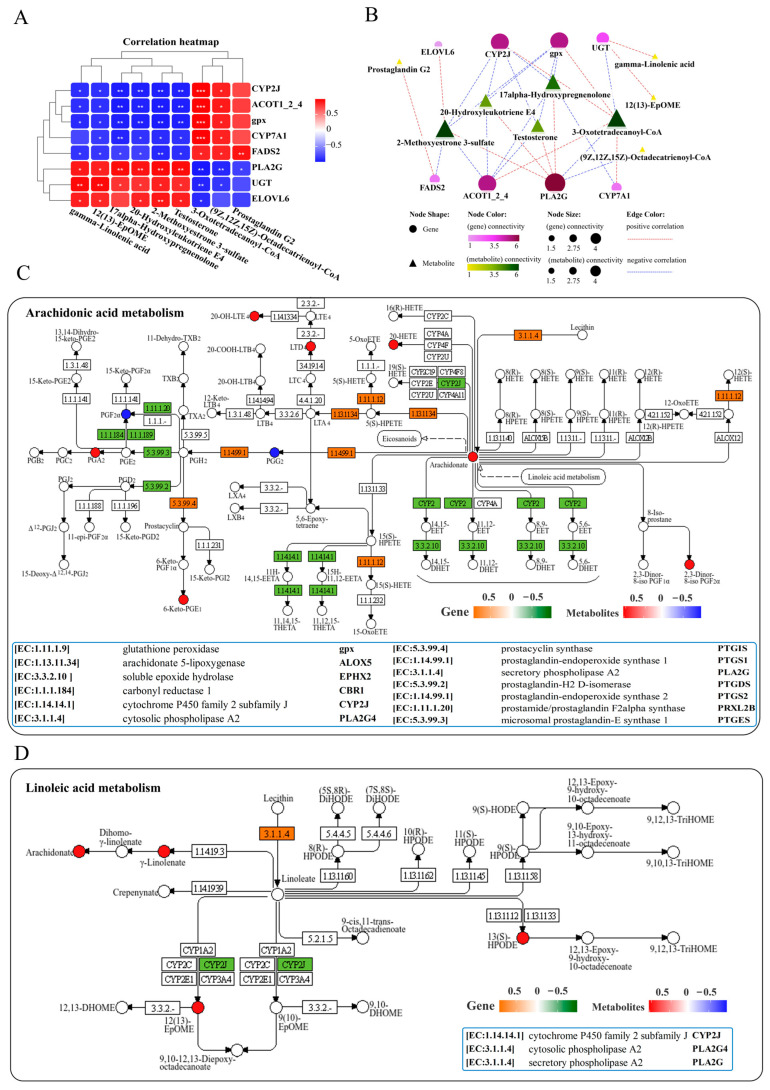
Joint analysis of DEGs and DMs in lipid metabolism pathways. (**A**) Correlation heatmap of DEGs and DMs. The red or blue squares illustrate the positive or negative correlation between DEGs and DMs, respectively. Asterisks in the boxes indicate a significant correlation between DEGs and DMs, and the number of asterisks indicate significance levels: * for 0.01 < *p* < 0.05, ** for 0.001 < *p* < 0.01, and *** for *p* ≤ 0.001. (**B**) Interaction network analysis of most significantly enriched DEGs and DMs in lipid metabolism pathways in the liver of largemouth bass. The orange dotted lines show a positive correlation between DEGs and DMs, while the blue dotted lines reveal a negative correlation between DEGs and DMs. (**C**) The DEGs and DMs in the arachidonic acid metabolism pathway are mapped to the KEGG pathway diagram. (**D**) The DEGs and DMs in the linoleic acid metabolism pathway are mapped to the KEGG pathway diagram. The orange or green box indicates significant upregulation or downregulation of the gene, respectively. The red or blue circles represent significant upregulation or downregulation of the metabolite, respectively.

Analysis of lipid metabolism in the liver resulted in a correlation heatmap showing the correlation between lipid metabolism-related genes and metabolites. The heatmap displays a network consisting of 8 key DEGs and 9 key DMs, showing positive or negative correlations among them (Figure 6A). Further interaction network analysis revealed a lipid metabolism regulatory network composed of 8 core gene nodes, including *CYP2J* (*cytochrome P450 family 2 subfamily J*), *PLA2G* (*secretory phospholipase A2*), *CYP7A1* (*cholesterol 7alpha-monooxygenase*), *UGT* (*glucuronosyltransferase*), *ACOT1_2_4* (*acyl-coenzyme A thioesterase 1/2/4*), *ELOVL6* (*elongation of very long chain fatty acids protein 6*), *FADS2* (*acyl-* *CoA 6-desaturase*) and *gpx*, as well as 9 core metabolite nodes (gamma-linolenic acid, 12(13)-EpOME, 17-alpha-hydroxypre-gnenolone, 2-methoxyestrone 3-sulfate, testosterone, 3-oxotetradeca-noyl-CoA, (9Z, 12Z, 15Z)-octadecatrienoyl-CoA, 20-hydroxyleukotriene E4 and prostaglandin G2) (Figure 6B). The genes *PLA2G* and *CYP2J*, as well as metabolites of 3-oxotetradecanoyl-CoA and 2-methoxyestrone 3-sulfate, showed the strongest correlations with other genes and metabolites (Figure 6B). KEGG pathway analysis indicated that infection with *N. seriolae* significantly altered arachidonic acid and linoleic acid metabolism (Figure 6C,D). Arachidonic acid metabolism was found to be significantly enriched in lipid metabolism, including DMs of 2,3-dinor-8-iso prostaglandin F2alpha, prostaglandin F2alpha, 20-hydroxyleukotriene E4, prostaglandin G2, and DEGs *PTGIS* (*prostacyclin synthase*), *PLA2G*, and *PRXL2B* (*prostamide/prostaglandin F2alpha synthase*). Meanwhile, the genes *PLA2G* [EC:3.1.1.4] and *CYP2J* were significantly altered in linoleic acid metabolism (Figure 6D).

### 3.7. Carbohydrate-Related Metabolic Changes in the Liver

**Figure 7:**
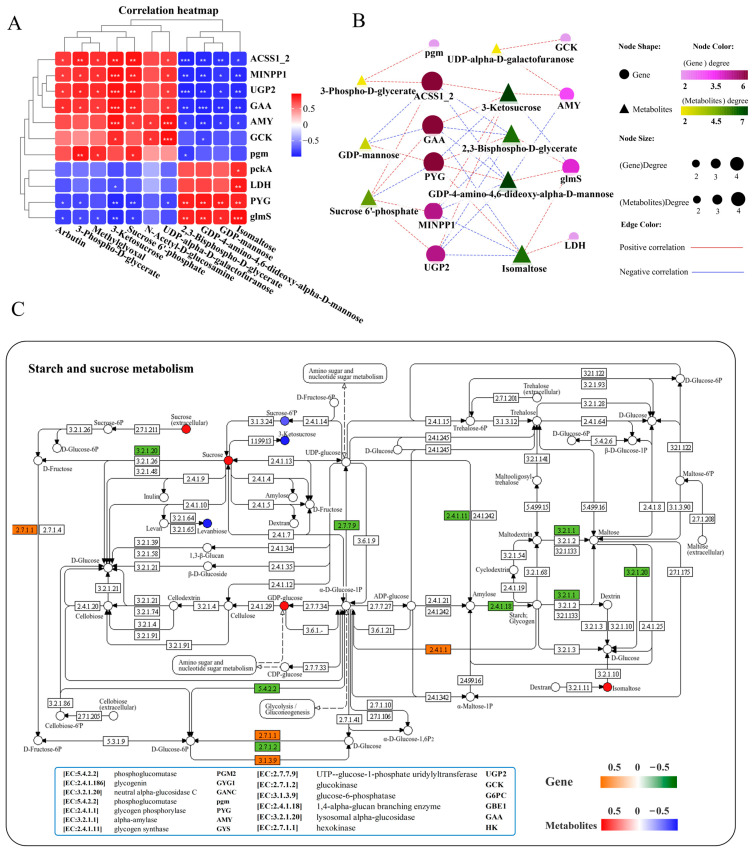
Joint analysis of DEGs and DMs in carbohydrate metabolism pathways. (**A**) Correlation heatmap of DEGs and DMs. The red or blue squares illustrate the positive or negative correlation between DEGs and DMs, respectively. Asterisks in the boxes indicate a significant correlation between DEGs and DMs, and the number of asterisks indicate significance levels: * for 0.01 < *p* < 0.05, ** for 0.001 < *p* < 0.01, and *** for *p* ≤ 0.001. (**B**) Interaction network analysis of most significantly enriched DEGs and DMs in carbohydrate metabolism pathways in the liver of largemouth bass. The orange dotted lines showed a positive correlation between DEGs and DMs, while the blue dotted lines revealed a negative correlation between DEGs and DMs. (**C**) The DEGs and DMs in the starch and sucrose metabolism pathway were mapped to the KEGG pathway diagram. The orange or green boxes indicate significant upregulation or downregulation of the gene, respectively. The red or blue circles represent significant upregulation or downregulation of the metabolite, respectively.

A correlation heatmap was used to analyze the correlation between carbohydrate metabolism-related DEGs and DMs, and the results showed a network composed of 11 key genes and 11 key metabolites that exhibited positive or negative correlations with each other (Figure 7A). Further interaction network analysis revealed a carbohydrate metabolism regulatory network consisting of 10 core gene nodes, including *ACSS1_2* (*acetyl- CoA synthetase*), *pgm* (*phosphoglucomutase*), *MINPP1* (*multiple inositol-polyphosphate phosphatase*), *LDH* (*l-lactate dehydrogenase*), *PYG* (*glycogen phosphorylase*), *UGP2* (*UTP-glucose-1-phosphate uridylyltransferase*), *GCK* (*glucokinase*), *glmS* (*methylaspartate mutase sigma subunit*), *AMY* (*alpha-amylase*), and *GAA* (*lysosomal alpha-glucosidase*), as well as 8 core metabolite nodes (3-phospho-D-glycerate, 2,3-bisphospho-D-glycerate, 3-ketosucrose, isomaltose, sucrose 6′-phosphate, GDP-4-amino-4,6-dideoxy-alpha-D-mannose, UDP-alpha-D-galactofuranose, GDP-mannose) (Figure 7B). Among them, genes *ACSS1_2*, *GAA*, and *PYG*, as well as metabolites 3-ketosucrose and GDP-4-amino-4,6-dideoxy-alpha-D-mannose, exhibited the highest correlations with genes and metabolites in the network (Figure 7B). ACSS1_2 (also known as acetyl-CoA synthetase) occupies an important position in cellular metabolism both in prokaryotic and eukaryotic cells [48]. Transcriptome analysis showed that the mRNA expression levels of *ACSS1_2* (EC: 6.2.1.1) and *GAA* (EC: 3.2.1.20) were significantly decreased, whereas *PYG* (EC: 2.4.1.1) markedly increased in the liver after infection (Figure 7C, Supplementary Figure S4). The *GAA*, *PYG*, and 3-ketosucrose were most strongly associated with the rest of the DEGs and DMs in the network diagram (Figure 7B), mainly enriched in the starch and sucrose metabolism pathways (Figure 7C). KEGG pathway analysis showed that infection with *N. seriolae* significantly altered the starch and sucrose metabolism pathways (Figure 7C) as well as glycolysis/gluconeogenesis (Supplementary Figure S4).

### 3.8. Arginine Suppresses Apoptosis Genes and Enhances Antioxidant Gene Expression

**Figure 8:**
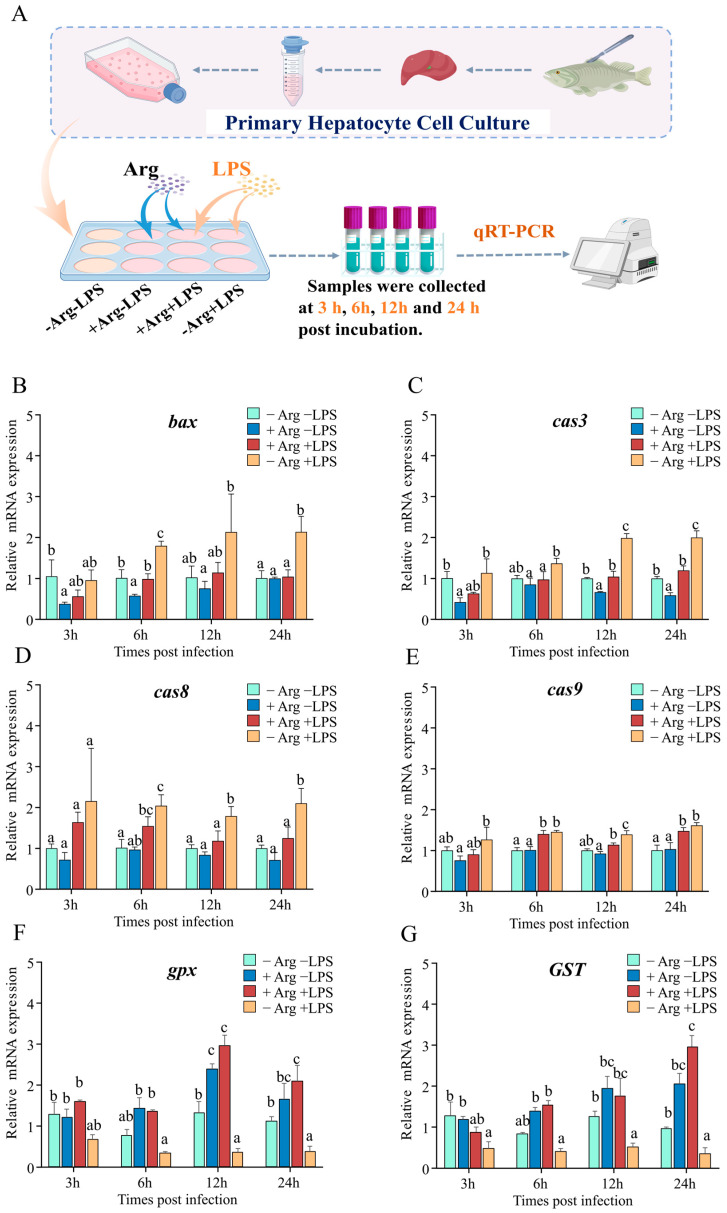
Regulation of apoptosis- and antioxidant-related gene expression in hepatocytes by co-incubation with arginine and LPS (n = 3). (**A**) Schematic diagram of arginine supplementation and LPS stimulation in hepatocytes. (**B**–**G**) Effects of arginine and LPS on the mRNA expression of apoptosis-related genes, including *bax* (**B**), *cas3* (**C**), *cas8* (**D**), *cas9* (**E**), and antioxidant-related genes, *gpx* (**F**) and *GST* (**G**), at 3, 6, 12, and 24 hpi. Data are presented as mean ± standard deviation (SD) and were analyzed using one-way ANOVA. Different letters indicate statistically significant differences (*p* < 0.05) among groups.

To investigate arginine’s effect on hepatocyte apoptosis, hepatocytes were incubated with varying concentrations of arginine (0 mM for the arginine-deficient group, 0.4 mM for the arginine-supplemented group) in vitro. As depicted in Figure 8A, arginine and LPS were added in different combinations, and cells were collected at 3, 6, 12, and 24 hpi for subsequent analysis. The results indicated that at 3 and 6 hpi, compared to the −Arg −LPS group, treatment with arginine alone (+Arg −LPS) significantly reduced *bcl-2-associated x protein* (*bax*) expression, whereas no significant differences were observed at 12 and 24 hpi. Under arginine-deficient conditions, LPS alone (−Arg +LPS) increased *bax* expression at 6 and 24 hpi, while arginine supplementation (+Arg +LPS) reduced *bax* expression (Figure 8B). Concurrently, compared to the −Arg −LPS group, treatment with arginine alone significantly reduced *cas3* expression at 3, 12, and 24 hpi. Under LPS stimulation alone, *cas3* expression increased at 12 and 24 hpi, whereas arginine supplementation significantly reduced *cas3* expression between 6 and 24 hpi (Figure 8C). Furthermore, under arginine-deficient conditions, LPS alone induced the expression of *cas8* and *cas9* between 6 and 24 hpi, while arginine supplementation reduced *cas8* expression at 12 and 24 hpi and *cas9* expression only at 12 hpi (Figure 8D,E). Regarding antioxidant-related genes, compared to the -Arg -LPS group, arginine treatment alone (+Arg −LPS) significantly increased *gpx* expression after 12 hpi, and this upregulation persisted at other time points. Conversely, LPS stimulation alone (−Arg +LPS) significantly reduced *gpx* and *GST* expression at 3, 12, and 24 hpi. Arginine supplementation in the presence of LPS (+Arg +LPS) significantly upregulated *gpx* and *GST* expression at 6, 12, and 24 hpi compared to LPS stimulation alone (−Arg +LPS), although no significant difference was observed at 3 hpi (Figure 8F,G).

## 4. Discussion

The liver of teleosts plays a dual role in metabolism and immune regulation, integrating the host’s defense and metabolic regulation functions [5,9]. *N. seriolae*, an intracellular pathogenic bacterium, can cause granulomatous lesions in target organs such as the liver after invading the host [13,22]. To further explore the strategies of the liver in coordinating metabolism and immune function following pathogen infection, we established a model of largemouth bass (*M. salmoides*) infected with *N. seriolae*. We first detected the pathological changes of the liver at different time points after *N. seriolae* infection and the transcription levels of apoptotic and immune-related genes. The joint analysis of transcriptome and metabolome identified key genes and metabolic pathways in the liver following *N. seriolae* infection and showed the response strategy of the liver post-infection.

Histological analysis effectively and intuitively characterizes pathological changes in organs following *N. seriolae* infection. A considerable number of white nodules were found in the abdominal cavity, spleen, and liver of the largemouth bass 7 days after infection with *N. seriolae*. By H&E staining, we found necrosis of cells around granulomas in the liver of diseased fish and accompanying infiltration of leukocytes on days 7 and 14 after infection with *N. seriolae*. This result is consistent with the typical clinical signs of *Nocardiosis* disease previously reported [13,22]. Furthermore, the transcriptional patterns of apoptotic and immune-related genes (inflammatory genes and antibacterial genes) in the liver were further examined by qPCR. The results showed that the expression levels of apoptotic (*bax*, *cas3*, and *cas9*), inflammatory (*tnfa*, *il8*, and *il10*), and antibacterial (*hep1*) genes peaked on day 2, and the expression of *hep2* significantly increased on day 4 and continued until day 14 post-infection. Under stress conditions (heat stress, pathogen infection), apoptosis in the liver of fish was accompanied by an increase in the transcription levels of apoptotic genes (e.g., *cas3*, *cas8*, and *cas9* genes) [10,49]. In our study, the expression of *bax*, *cas3*, and *cas9* genes in the liver significantly increased post-*N. seriolae* infection, although the expression of *cas8* did not change. Pathogen infection could trigger the host inflammatory response, with immune cells secreting a high number of cytokines involved in regulating host inflammation, and at the same time, the expression of inflammatory gene products increases considerably [50]. Our results demonstrated that *tnfa* and *il8* gene expression were considerably increased in the liver of largemouth bass following infection with *N. seriolae*, which was comparable to previously published findings [51]. IL-10 is a cytokine with anti-inflammatory functions that restricts the inflammatory response to infection and minimizes tissue damage [52]. Here, we detected a significant increase in the expression of *il10* in the liver, indicating that the tissue repair of largemouth bass was initiated following *N. seriolae* infection.

In addition to inflammatory and apoptotic factors, the complement system plays a crucial immunoregulatory role in teleost fish, involving host immune defense and blood clotting. Moreover, the complement system is a crucial component of both innate and adaptive immunity [53]. The complement system in teleosts could be triggered by three pathways, namely the classical pathway, the alternative pathway, and the lectin pathway [54,55]. These pathways ultimately lead to the lysis and activation of complement components, producing a series of biologically active products such as C1q, C3a, C3b, C5a, and C5b [56]. In Nile tilapia, the expression of OnC1qs in the liver is significantly upregulated post-challenge by LPS and *Streptococcus agalactiae*, and recombinant C1qs enhanced the expression of inflammatory factors (IL-6, IL-8, and IL-10) in head kidney adherent leukocytes [57]. In black rockfish (*Sebastes schlegelii*), the C1qDC protein exhibited antibacterial activity against *Vibrio parahaemolyticus* by enhancing the phagocytic activity of macrophages to increase the resistance of fish to bacterial infection [58]. Poc1ql3 could protect Japanese flounder (*Paralichthys olivaceus*) against *Edwardsiella tarda* infection [59]. PoC3 in the serum of Japanese flounder has the ability to bind to a variety of bacteria (e.g., *Pseudomonas fluorescens*, *Vibrio anguillarum*, *Edwardsiella tarda*, *Vibrio harveyi*, *Streptococcus iniae*, and *Escherichia coli*), and rPoC3a mediated increased resistance against bacterial infection [60]. In grass carp (*Ctenopharyngodon idella*), C3a significantly enhances the phagocytic activity of IgM^+^ B cells [61]. The coagulation cascade is the process of forming blood clots to prevent bleeding and vascular rupture in the body [62]. Our findings revealed that the mRNA expression levels of inflammatory genes increased post-infection, and the complement and coagulation cascade pathways were activated in the liver of largemouth bass. These data indicated that the complement system is involved in regulating the inflammation response in largemouth bass liver and the process of resistance to *N. seriolae* infection. Our data offers a foundation for future study into how the complement system governs largemouth bass tolerance to bacterial infection and the immune response in the liver. Further research is needed on the regulatory mechanism of the complement system in largemouth bass against *N. seriolae* infection.

The liver plays a crucial role in metabolism [24], and alterations in liver metabolites following pathogen infection could indicate the liver’s response strategy. The metabolic results revealed that DMs are primarily enriched in pathways related to amino acid metabolism, including glutathione, arginine, proline, glycine, serine, and threonine metabolism, as well as carbohydrate and lipid metabolism pathways. The findings indicated that pathogen infection has an expansive impact on liver metabolism. The joint analysis of transcriptome and metabolome provided insight into the underlying mechanisms of biological processes that cannot be identified employing a single-omic method [24,63]. Then, further joint analysis of transcriptome and metabolome in the liver showed that the genes and metabolites that commonly respond are mainly enriched in the following metabolic pathways: protein digestion and absorption, glycolysis/gluconeogenesis, glycerophospholipid metabolism, and arachidonic acid metabolism. Different metabolic pathways are closely related, as shown in the Sankey diagram of the flow of biological information, which includes pathways related to amino acid metabolism such as glycine, serine, and threonine metabolism, arginine and proline metabolism, tryptophan metabolism, carbohydrate metabolism related to glycolysis/gluconeogenesis, and lipid metabolism related to arachidonic acid metabolism, biosynthesis of unsaturated fatty acids, and arachidonic acid metabolism. These results suggest that there are correlations among genes, metabolites, and metabolic pathways involved in the metabolism of substances in the liver post-*N. seriolae* infection.

Amino acid metabolism is critical for the survival of all organisms, as it can supply energy and intermediate metabolites for cellular metabolism and promote cytokine secretion to regulate the body’s immune response [64,65]. The infection with *N. seriolae* changed the amino acid metabolism in the liver of largemouth bass, particularly arginine, proline, glutathione, glycine, serine, and threonine metabolism. In mammals, arginine is a key immunomodulator [65]. In fishes, arginine is crucial for regulating the inflammatory response, immune response, and disease resistance [66,67]. Additionally, arginine can enhance the phagocytic capacity and bactericidal activity of fish macrophages to improve the nonspecific immune response of the host [68,69]. Serine can regulate macrophage polarization, increase the transcription of *il1β*, and trigger inflammation [70]. Its absence inhibits the transcription of *il1β* and the polarization of pro-inflammatory macrophages [71]. According to the results, it can be inferred that the liver regulated the host’s inflammatory response and immune strategy by altering the metabolism of amino acids in response to pathogen infection.

Furthermore, glutathione metabolism is critical to the endogenous antioxidant system in fishes. Glutathione, its key product, functions as an endogenous antioxidant and plays a significant role in preventing oxidative stress, scavenging free radicals, and mitigating biological damage [72]. Glutathione-S-transferase (GST), a crucial component of the antioxidant enzyme system, is vital for eliminating free radicals and reducing biological damage, thereby alleviating oxidative stress damage. In this study, key genes involved in glutathione metabolism exhibited an overall active expression pattern following pathogenic stimulation. However, the constructed regulatory network for glutathione metabolism indicated a downregulated pattern for key genes *gpx* and *GST*, which directly participate in glutathione recycling and redox balance. The concomitant downregulation of GSH reflects a compromised antioxidant capacity in the liver, as reduced *gpx* and *GST* activity would impair the reduction of oxidized glutathione to GSH. This metabolic shift likely exacerbates oxidative damage during *N. seriolae* infection. Importantly, arginine supplementation significantly increased *gpx* and *GST* expression in liver cells under LPS stimulation. Previous studies suggested that arginine, as a substrate for glutamate synthesis [73,74,75], likely stimulates endogenous GSH synthesis [76]. The upregulation of *gpx* and *GST* by arginine may help restore glutathione homeostasis, thereby enhancing the liver’s ability to counteract oxidative stress. On the basis of the results of this study, we hypothesize that arginine may regulate GSH synthesis, thereby exerting an antioxidant effect in the largemouth bass liver, although the specific mechanisms warrant further investigation.

Integrated transcriptomic and metabolomic analyses revealed significant regulation of lipid metabolism, with pronounced modulation of *PLA2G* and *CYP2J* expression. The *PLA2G* and *CYP2J* genes are not only involved in arachidonic acid metabolism but also play crucial regulatory roles in the linoleic acid metabolism pathway. PLA2G is also known as sPLA2 (secretory phospholipase A2). PLA2 belongs to the family of phospholipase enzymes that hydrolyze the ester bond at the sn-2 position of the phospholipids, resulting in the production of a free fatty acid (such as arachidonic acid and oleic acid) and a lysophospholipid [77]. Arachidonic acid can be processed into inflammatory leukotrienes by cyclooxygenases (COXs), lipoxygenases (LOXs), and cytochrome P450 (CYP450) enzymes, hence enhancing local inflammatory responses [78]. SPLA-IIA is also known as “inflammatory sPLA2” since it was released by various cells (e.g., macrophages, monocytes, T cells, mast cells, and neutrophils) in response to inflammatory reactions [79,80]. In humans, it has been reported that CYP2J2 (cytochrome P450 family 2 subfamily J 2) has various functions, including anti-inflammatory effects [81], metabolic promotion [82], and immune regulation [83]. Under pathological conditions, CYP2J2 and the metabolites derived from arachidonic acid play key roles in regulating cardiovascular function and malignant tumors [82]. Our findings show that the transcription level of PLA2G is significantly increased in the enriched arachidonic acid metabolism pathway, indicating that PLA2 in the liver promotes phospholipid hydrolysis and that arachidonic acid is further processed into inflammatory leukotrienes, inducing an inflammatory response in the liver following *N. seriolae* infection.

Acetyl-CoA is a crucial energy source involved in the tricarboxylic acid cycle, fatty acid synthesis, cholesterol production, and histone acetylation [84,85]. Acetyl-CoA synthetase catalyzes the conversion of acetate into acetyl-CoA [48]. The glycolytic pathway produces pyruvate, which enters the mitochondria and is catalyzed by the pyruvate dehydrogenase complex to produce NADH, CO_2_, and acetyl-CoA [86]. Subsequently, acetyl-CoA enters the tricarboxylic acid cycle, which is an essential metabolic pathway in cells that completely oxidizes organic compounds to CO_2_ and H_2_O, releasing energy [87]. The transcription level of the acetyl-CoA synthetase gene was significantly decreased, indicating that bacterial infection significantly altered the liver′s energy metabolism and disrupted the metabolic balance of the liver. Changes in 3-ketosucrose metabolite content and *GAA* and *PYG* gene expression imply that starch and sucrose metabolism is involved in the immune response strategies in the liver. Taken together, these findings demonstrated that the invasion of *N. seriolae* drastically alters the starch and sucrose metabolism, as well as glycolysis/gluconeogenesis pathways in the liver of largemouth bass, disrupting the energy metabolism balance in the liver.

Integrated-omics analyses revealed that amino acid metabolism, particularly arginine metabolism and glutathione metabolism, responds to *N. seriolae* infection, identifying arginine and glutathione as key metabolites involved in the regulation of inflammation, immune responses, antioxidant processes, and disease resistance [65,66,67,88,89,90]. Therefore, to further investigate the regulatory effects of arginine on inflammation and apoptosis in largemouth bass hepatocytes, an in vitro LPS-stimulation model was established. LPS stimulation significantly induced the expression of apoptosis-related genes, consistent with previous studies [91,92,93]. Caspases, members of the cysteine protease family, are crucial components of the apoptotic pathway [94,95]. Among these, cas8 and cas9 function as initiators, playing significant roles in the apoptotic response, whereas cas3, an executioner caspase, is essential for cellular breakdown and apoptotic body formation [96]. This study’s results demonstrated that, at specific time points, LPS induction alone significantly upregulated the expression of apoptosis-related genes (*bax*, *cas3*, *cas8*, and *cas9*). However, with arginine supplementation (+Arg +LPS), the expression levels of these genes were significantly reduced, indicating that L-arginine can effectively inhibit LPS-induced apoptosis, consistent with previous in vivo findings [97]. Notably, compared to LPS stimulation alone, arginine supplementation (+Arg +LPS) resulted in a more pronounced downregulation of *cas3* expression across most time points (6 to 24 hpi), potentially due to its crucial role as an executioner caspase in the cellular breakdown and apoptotic body formation. Furthermore, external stressors (e.g., pathogen infection, heat stress, or pesticide residues) can increase the expression of apoptosis-related genes in largemouth bass [8,49,98]. Regarding key antioxidant genes, arginine supplementation significantly enhanced the expression of antioxidant genes (*gpx* and *GST*) under LPS stimulation at 6, 12, and 24 hpi. Previous studies have shown that arginine plays an important role in alleviating oxidative stress and enhancing immune function [65,88,89,90]. The findings of this study suggest that arginine supplementation helps organisms resist external stress, enhance antioxidant capacity, and promote overall health.

Our in vitro experiments utilized 0.4 mM arginine to demonstrate its immunomodulatory effects. However, translating this dosage into practical aquaculture applications requires careful consideration. In fish nutrition studies, dietary arginine supplementation typically ranges from 3.0% to 8.1% of feed, depending on species and life stage. While appropriate supplementation can enhance physiological functions, excessive intake may disrupt amino acid balance or induce metabolic stress [99,100]. In *M. salmoides*, supplementation with 2.84% arginine significantly increased total nitric oxide synthase (T-NOS) activity and antioxidant capacity [101]. However, arginine and lysine share cellular transport mechanisms, leading to competition for transporters [102,103]. Excessive arginine intake may disturb the arginine-lysine antagonistic balance, potentially affecting metabolic homeostasis [104,105]. Beyond its antioxidant role, arginine metabolism in teleost hepatocytes involves several key pathways with trade-offs. Arginine is the sole precursor for nitric oxide (NO) synthesis, catalyzed by nitric oxide synthase (NOS), playing a crucial role in macrophage activation and pathogen clearance [101,106]. However, excessive NO can react with superoxide radicals to form peroxynitrite, exacerbating oxidative stress and tissue damage [107]. Additionally, arginine metabolism via ornithine decarboxylase (ODC) produces polyamines such as putrescine, spermidine, and spermine, which support cell development and protein synthesis [108,109]. Under hypoxic conditions, increased arginase and ODC activity suggests a potential role for arginine in stress adaptation [110]. Notably, fish primarily rely on ammoniotelism and exhibit limited urea cycle activity [111]. Excessive arginine supplementation may directly stimulate arginase activity, accelerating its metabolism and excretion as nitrogenous waste [101], thereby restricting its flux into urea synthesis. These dual roles highlight the delicate balance of dietary arginine supplementation: while appropriate levels enhance immunity and antioxidant defense, excessive intake may increase oxidative stress risks. Future studies should validate these findings through feeding trials with graded arginine levels. Optimizing arginine concentrations in aquaculture feed requires a comprehensive understanding of these metabolic interactions to maximize health benefits.

## 5. Conclusions

This study aimed to investigate the strategies of liver metabolism and immune response in largemouth bass (*M. salmoides*) following infection with *N. seriolae*. Our findings revealed that pathogen infection caused severe pathological changes in the liver and elicited the transcription of genes related to apoptosis, inflammation, and antibacterial infection. Integrative transcriptomic and metabolomic analyses identified numerous DEGs and DMs, revealing that *N. seriolae* infection significantly altered amino acid metabolism (arginine and proline metabolism, glutathione metabolism), lipid metabolism (arachidonic acid metabolism), and carbohydrate metabolism (starch and sucrose metabolism, glycolysis/gluconeogenesis) in the largemouth bass liver. In-vitro incubation of liver cells with LPS and arginine showed that arginine supplementation significantly reduced the expression of LPS-induced apoptosis-related genes and notably increased the expression of antioxidant-related genes. This study explored the immune strategies and metabolic regulation of the liver after *N. seriolae* infection and clarified the anti-apoptotic and antioxidant regulatory functions of arginine. However, the mechanisms underlying arginine regulation of immune responses and mitigation of oxidative stress warrant further investigation, providing a foundation for future sustainable aquaculture practices.

## Data Availability

The data presented in this study are available on request from the corresponding author.

## Supplementary Materials

The following supporting information can be downloaded at: https://www.mdpi.com/article/10.3390/antiox14060681/s1, Figure S1: Symptoms of largemouth bass infected with *N.Seriola* at 7dpi; Figure S2: GSEA for the KEGG (A) proteasome pathway, (B) amino sugar and nucleotide sugar metabolism pathway, (C) glutathione metabolism (D) glycolysis/gluconeogenesis pathway, (E) pentose and glucuronate interconversion pathway, (F) complement and coagulation cascades pathway, and (G) tryptophan metabolism pathway; Figure S3: The DEGs and DMs in the glycine, serine, and threonine metabolism pathway were mapped to the KEGG pathway diagram; Figure S4: The DEGs and DMs in the glycolysis/gluconeogenesis were mapped to the KEGG pathway diagram; Table S1: Primers used in this study.

## Abbreviations

DEGs	Differentially expressed genes	gpx	glutathione peroxidase
DMs	Differentially expressed metabolites	GST	glutathione-S-transferase
KEGG	Kyoto Encyclopedia of Genes and Genomes	CYP1A1	cytochrome p450 family 1 subfamily a1
GO	Gene Ontology	DHKTD1	2-oxoadipate dehydrogenase E1 component
GSEA	Gene Set Enrichment Analysis	ALDH	aldehyde dehydrogenase (NAD+)
OPLS-DA	orthogonal partial least squares discriminant analysis	HNMT	histamine n-methyltransferase
VIP	variable importance in the projection	ahcY	adenosylhomocysteinase
DMEM	Dulbecco’s Modified Eagle Medium	glyA	glycine hydroxymethyltransferase
PS	penicillin-streptomycin	serB	phosphoserine phosphatase
FBS	fetal bovine serum	GLDC	glycine dehydrogenase
LPS	lipopolysaccharide	TDO2	tryptophan 2,3-dioxygenase
H&E	hematoxylin and eosin	TST	thiosulfate/3-mercaptopyruvate sulfurtransferase
qPCR	quantitative polymerase chain reaction	DAO	d-amino-acid oxidase
LC-MS	liquid chromatography-mass spectrometry	PGAM	2,3-bisphosphoglycerate-dependent phosphoglycerate mutase
GSH	glutathione	Lipid metabolism-related genes/protein	
Arg	arginine	CYP2J	cytochrome P450 family 2 subfamily J
Immune and antioxidant-related genes		PLA2G	secretory phospholipase A2
*bax*	bcl-2-associated x protein	CYP7A1	cholesterol 7alpha-monooxygenase
*cas3/8/9*	caspase-3/8/9	UGT	glucuronosyltransferase
*tnfa*	tumor necrosis factor-alpha	ACOT1_2_4	acyl-coenzyme A thioesterase 1/2/4
*il8/il10*	interleukin-8/10	ELOVL6	elongation of very long chain fatty acids protein 6
*tgfβ*	transforming growth factor beta	FADS2	acyl- CoA 6-desaturase
*hepl/hep2*	hepcidin-1/2	Carbohydrate metabolism-related genes/protein	
*lyso*	lysozyme	ACSS1_2	acetyl- CoA synthetase
*pis*	piscidin	pgm	phosphoglucomutase
*sod1*	superoxide dismutase 1	MINPP1	multiple inositol-polyphosphate phosphatase
*nod1*	nucleotide-binding oligomerization domain-containing protein 1	LDH	l-lactate dehydrogenase
*odc1*	ornithine decarboxylase 1	PYG	glycogen phosphorylase
*arg2*	arginase 2	UGP2	UTP-glucose-1-phosphate uridylyltransferase
*c7b*	complement component 7b	GCK	glucokinase
*ass1*	argininosuccinate synthase 1	glmS	methylaspartate mutase sigma subunit
*asl*	argininosuccinate lyase	AMY	alpha-amylase
*myd88*	myeloid differentiation primary response 88	GAA	lysosomal alpha-glucosidase
