# Supplementary material for: Arginine-Mediated Liver Immune Regulation and Antioxidant Defense in Largemouth Bass (Micropterus salmoides): Multi-Omics Insights into Metabolic Remodeling During Nocardia seriolae Infection
Source: Antioxidants (Basel). 2025 Jun 3;14(6):681. doi: 10.3390/antiox14060681 (PMC12189571; doi:10.3390/antiox14060681)
Supplement: Supplementary file 1 [file antioxidants-14-00681-s001.zip › antioxidants-3638563-supplementary.pdf]

Supplementary Figure S1. Symptoms of largemouth bass infected with *N.Seriola* at 7dpi. The red arrows indicated white nodules on the abdominal cavity, liver and spleen of the largemouth bass.

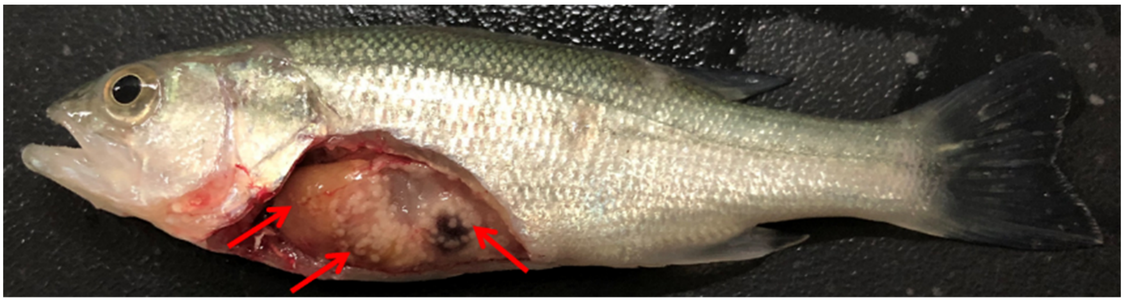

Supplementary Figure S2. GSEA for the KEGG (A) proteasome pathway, (B) amino sugar and nucleotide sugar metabolism pathway, (C) glutathione metabolism (D)glycolysis/gluconeogenesis pathway, (E) pentose and glucuronate interconversion pathway, (F) complement and coagulation cascades pathway, and (G) tryptophan metabolism pathway.

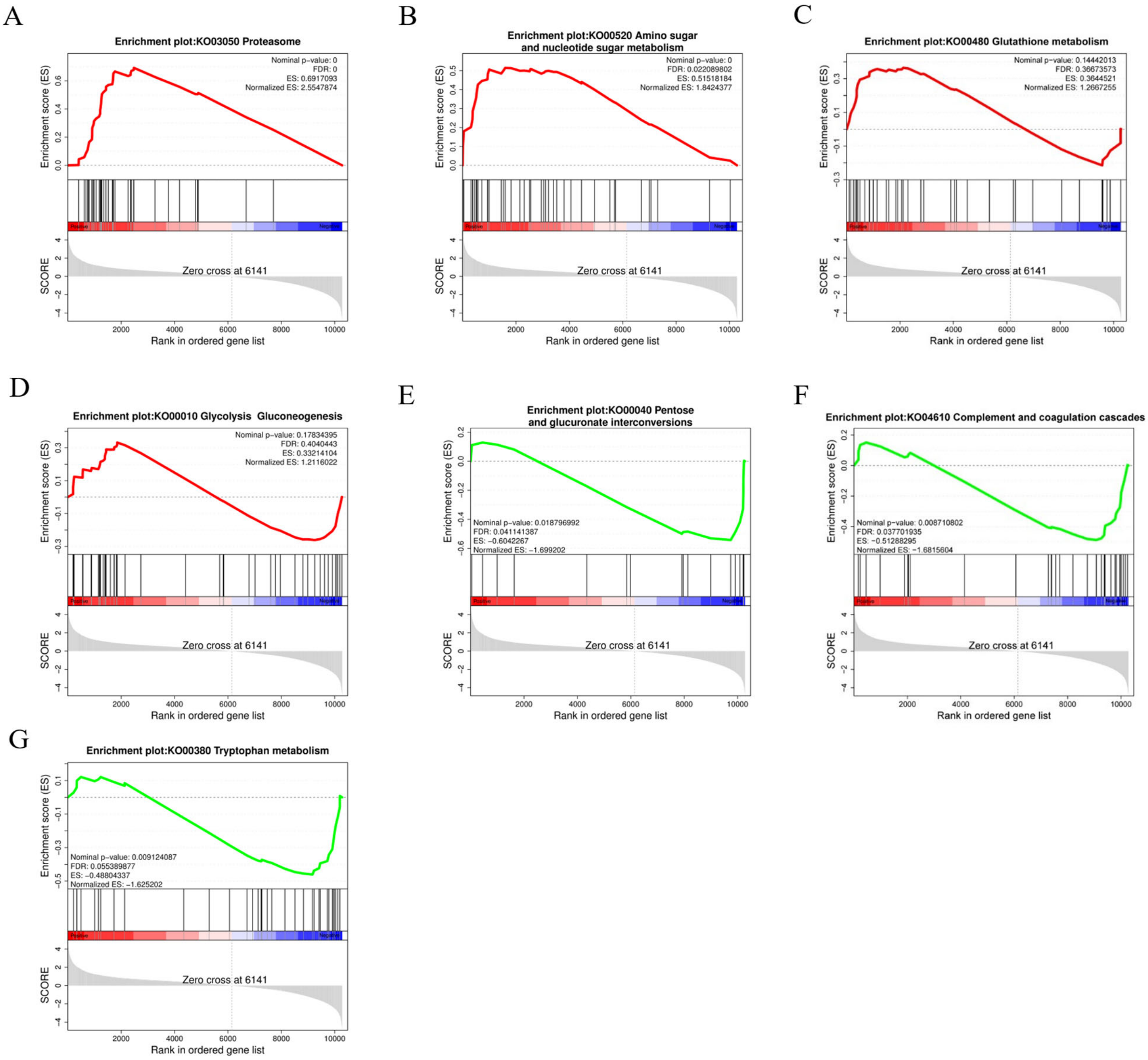

Supplementary Figure S3. The DEGs and DMs in the glycine, serine, and threonine metabolism pathway were mapped to the KEGG pathway diagram. The orange or green box indicated significant upregulation or downregulation of the gene, respectively. The red or blue circle represented significant upregulation or downregulation of the metabolite, respectively.

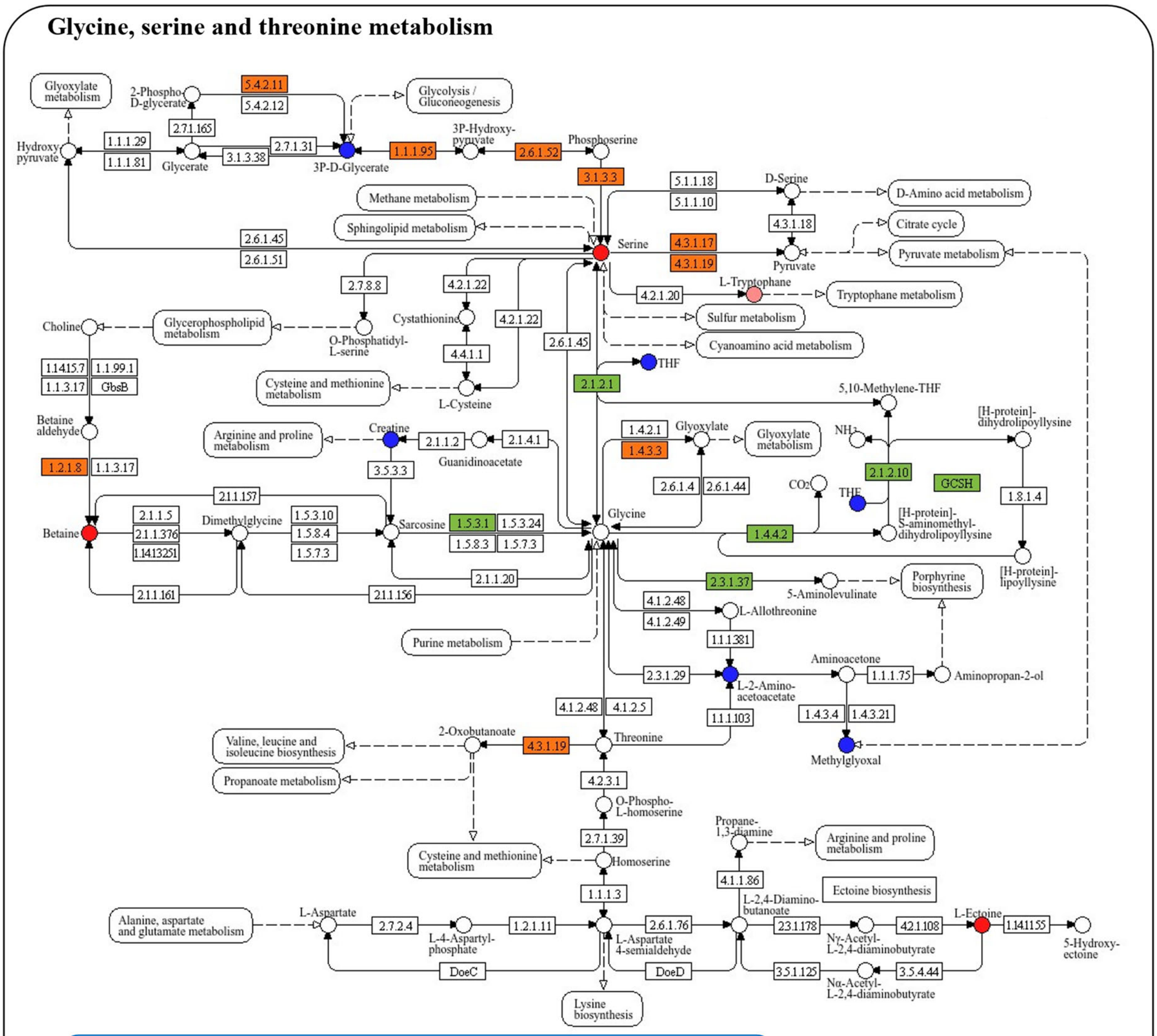

**[EC:1.1.1.95]**	D-3-phosphoglycerate dehydrogenase / 2-oxoglutarate reductase	**serA**
**[EC:2.1.2.1]**	glycine hydroxymethyltransferase	**glyA**
**[EC:1.2.1.8]**	aldehyde dehydrogenase family 7 member A1	**ALDH7A1**
**[EC:3.1.3.3]**	phosphoserine phosphatase	**serB**
**[EC:1.5.3.1]**	sarcosine oxidase / L-pipecolate oxidase	**PIPOX**
**[EC:2.6.1.52]**	phosphoserine aminotransferase	**serC**
**[EC:2.1.2.10]**	aminomethyltransferase	**gcvT**
**[EC:1.4.3.3]**	D-amino-acid oxidase	**DAO**
**[EC:2.3.1.37]**	5-aminolevulinate synthase	**ALAS**
**[EC:4.3.1.17 4.3.1.19]**	L-serine/L-threonine ammonia-lyase	**SDS, SDH, CHA1**
**[EC:5.4.2.11]**	2,3-bisphosphoglycerate-dependent phosphoglycerate mutase	**PGAM**
**[EC:1.4.4.2]**	glycine dehydrogenase	**GLDC**

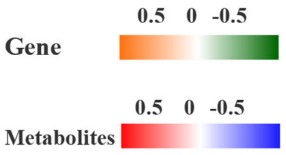

Supplementary Figure S4. The DEGs and DMs in the glycolysis/gluconeogenesis were mapped to the KEGG pathway diagram. The orange or green box indicated significant upregulation or downregulation of the gene, respectively. The red or blue circle represented significant upregulation or downregulation of the metabolite, respectively.

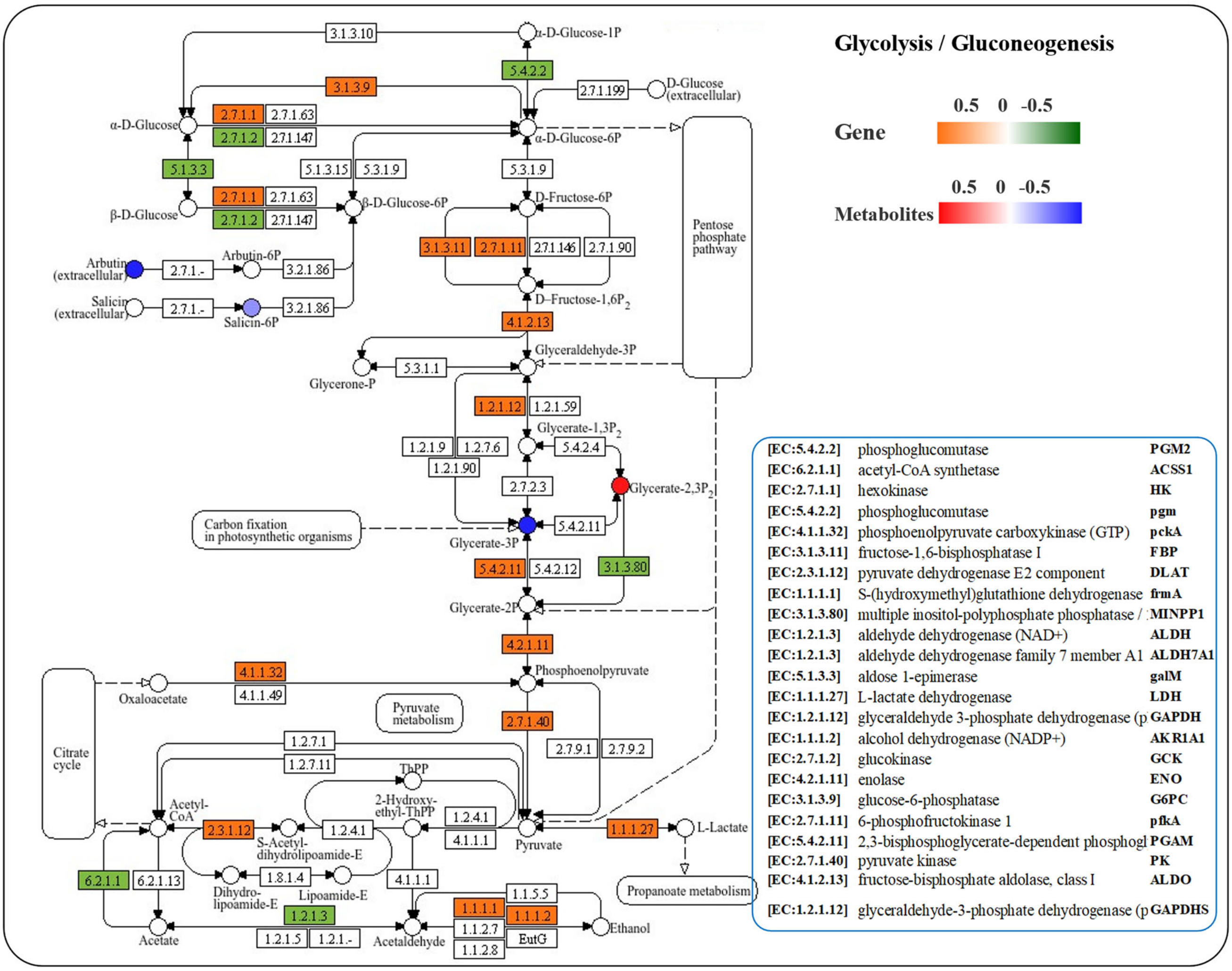

Supplemental Table S1. Primers used in this study.

Primer name	Primer sequence (5'-3')	Accession number
β-actin-F	GAGCGTGGCTACTCCTTCAC	XM_038695351.1
β-actin-R	GTCAGGCAGCTCGTAGCTCT	
bax-F	GCTGCTCAAGATTGCAGATG	XM_038701918.1
bax-R	GAAGAGAGCCACCACTCGAC	
cas3-F	GCTTCATTCGTCTGTGTTC	XM_038713063.1
cas3-R	CGAAAAAGTGATGTGAGGTA	
cas8-F	GAGACAGACAGCAGACAACCA	XM_038718636.1
cas8-R	TTCCATTTCAGCAAACACATC	
cas9-F	CTGGAATGCCTTCAGGAGACGGG	XM_038734900.1
cas9-R	GGGAGGGGCAAGACAACAGGGTG	
tnfα-F	CTAGTGAAGAACCAGATTGT	XM_038710731.1
tnfα-R	AGGAGACTCTGAACGATG	
il8-F	GCAGCAAAGTCATTGTCACC	MW751832.1
il8-R	TGGCAGGAATCAGCTCTACC	
il10-F	CGGCACAGAAATCCCAGAGC	XM_038696252.1
il10-R	CAGCAGGCTCACAAAATAAACATCT	
tgfβ-F	GCTCAAAGAGAGCGAGGATG	XM_038693206.1
tgfβ-R	TCCTCTACCATTCGCAATCC	
hep1-F	ACACTCGTGCTCGCCTTTAT	EU502754.1
hep1-R	CAAGGAGAGGTGGCTTTGAC	
hep2-F	CCGTCGTGCTCACCTTTATT	EU502755.1
hep2-R	CGCGCTTCTGTCTGTTGTTA	
pis-F	TATTGTGATCTTTCTCGTGTTGTCC	MT681907.1
pis-R	CTGCTCTTGGACACCGTGGT	
lyso-F	GTATAACGCCTGGGGACTGA	MH087462.1
lyso-R	AGGCTGCTATTCCTCCCTTC	
**sod1*-F	GGTTTCCATGTCCATGCTTT	MK614709.1
**sod1*-R	TGCGACATTATCTGCTCCTG	
**nod1*-F	GGTCCAAGCAAACACAGGTT	XM_038712292.1
**nod1*-R	GCTGGTTCCCTTTCACACAT	
**odc1*-F	AGGCAGTGTGTCGCCTAAGT	XM_038728782.1
**odc*1-R	CTGACACCGATCACATCCAG	
**arg2*-F	ACCTCAACTTCCACCACCTG	XM_038728622.1
**arg2*-R	CATGACAAGGGTGTGACCAG	
**c7b*-F	GTACGACAACTGGGCCTCAT	XM_038734682.1
**c7b*-R	CCTCTGTGGCTCTTTTCAGG	
**ass1*-F	ATGAGGACCGATACCTGCTG	XM_038733332.1
**ass1*-R	TCCATGGTGCGATGATCTTA	
**asl*-F	CATACAGCACAGGCAGCAGT	XM_038709368.1
**asl*-R	CATCATGAACCCTGCACAAC	
**gpx*-F	TCGTGAGAATGGCTGGGAATG	XM_038697220.1
**gpx*-R	ATAACGAGTCCCTTGGCAGTG	
**GST*-F	AAGGACATGACTCTGCTGTGG	XM_038724634.1
**GST*-R	CTGACCCCTGGGATTCATGTC	
**myd88*-F	TGGAGGAGGTGGAGAGAAAA	XM_038728827.1

**myd88*-R	GGACACAGCTGTCAACCTCA

cas3, *caspase-3*; *cas8*, *caspase-8*; *cas9*, *caspase-9*; *hep1*, *hepcidin-1*; *hep2*, *hepcidin-2*. *lyso*, *lysozome*; *pis*, *gpx*, *GST*, *piscidin*. *Indicates the isoform specific primers used for validating the differentially expressed genes identified by RNA-Seq.
